# Glycosylation Circuit Enables Improved Catalytic Properties for Recombinant Alkaline Phosphatase

**DOI:** 10.1021/acsomega.3c04669

**Published:** 2023-08-31

**Authors:** Eray Ulaş Bozkurt, İrem Niran Çağıl, Ebru Şahin Kehribar, Musa Efe Işılak, Urartu Özgür Şafak Şeker

**Affiliations:** UNAM- Institute of Materials Science and Nanotechnology, National Nanotechnology Research Center, Bilkent University, Ankara 06800, Turkey

## Abstract

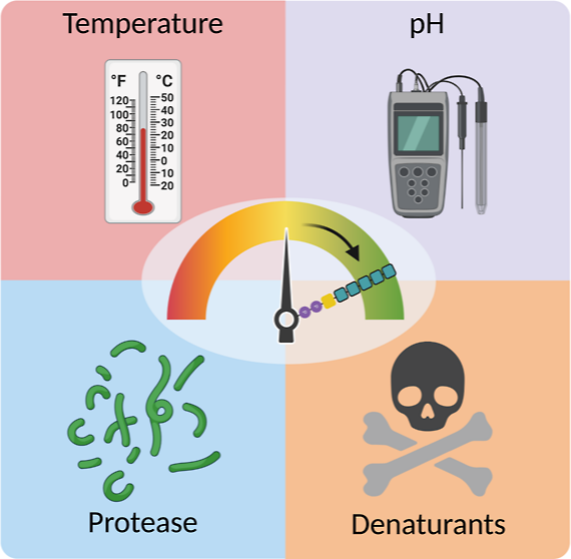

Protein glycosylation is one of the most crucial and common post-translational modifications. It plays a fate-determining role and can alter many properties of proteins. Here, we engineered a *Campylobacter jejuni* N-linked glycosylation machinery by overexpressing one of the core glycosylation-related enzymes, PgIB, to increase the glycosylation rate. It has been previously shown that by utilizing N-linked glycosylation, certain recombinant proteins have been furnished with improved features, such as stability and solubility. We utilized N-linked glycosylation using an engineered glycosylation pathway to glycosylate a model enzyme, the alkaline phosphatase (ALP) enzyme in *Escherichia coli*. We have investigated the effects of glycosylation on enzyme properties. Considering the glycosylation mechanism is highly dependent on accessibility of the glycosylation tag, ALP constructs carrying the glycosylation tag at different locations of the gene have been constructed, and glycosylation rates have been calculated. Our results showed that, upon glycosylation, ALP features in terms of thermostability, proteolytic stability, tolerance to suboptimal pH, and denaturing conditions are dramatically improved. The results indicated that the N-linked glycosylation mechanism can be employed for protein manipulation for industrial applications.

## Introduction

Protein glycosylation is one of the most prevalent, diverse, and crucial post-translational modifications.^1^ The phenomenon is found in all domains of life,^2^ and over 50% of eukaryotic proteomes is known to be glycosylated.^3^ Glycosylation plays a significant role in proteins’ fate, and it can alter many features of proteins, including their stability and activity, therefore attracting wide attention from the scientific community.^4,5^ Although recombinant proteins are frequently utilized in medical and industrial applications,^6^ many proteins often suffer from low stability under suboptimal thermal and chemical conditions and exhibit low solubility. They also may aggregate over time, which may cause decreased efficiency and increased immunogenicity. Such factors limit the efficient usage of recombinant proteins in many applications, and manipulation of these properties could create new opportunities in medical and industrial approaches.^7^

Protein glycosylation, once thought to be unique for eukaryotes, was later discovered in Bacteria and Archaea.^8^ Although glycosylation systems are more diverse in eukaryotes, many N-linked—addition of glycans to the nitrogen group of asparagine residues—and O-linked—addition of glycans to the hydroxyl oxygen of serine or threonine residues—glycosylation mechanisms are found in bacteria.^9^ N-Linked glycosylation was first described in *Campylobacter jejuni* (*C. jejuni*),^10^ and it was shown that *C. jejuni* utilizes the pgl (protein glycosylation) mechanism to attach glycan groups *en bloc* to more than 65 proteins in the periplasm.^11^ Glycosylated proteins play a role in contributing to the fitness of the bacterium in the gut and protecting it from proteases.^12,13^ This discovery speeded up the progress being made in the field, and many other findings followed.^3^ Today, it is known that N-linked glycosylation occurs in at least 49 species that possess the compounds of pgl pathway.^14^ Although there are more novel pathways discovered, such as the sequential N-linked glycosylation mechanism of *Haemophilus influenzae* (*H. influenzae*)^15^ and cytosolic glycosylation machinery of *Actinobacillus pleuropneımoniae*,^16^*C. jejuni*’s N-linked glycosylation remains to be the most extensively characterized pathway.^3^

*C. jejuni’s* N-linked glycosylation mechanism involves 13 genes encoding enzymes for the synthesis of glycan and *en bloc* transfer of the produced glycan moiety onto the acceptor protein. N-Glycan synthesis is initiated by the formation of uridine diphosphate (UDP)-activated-*N*-acetylglucosamine (UDP-GlcNAc) on the cytoplasmic side of the cell. The PglF enzyme, a C6 dehydratase, converts UDP-GlcNAc to UDP-2-acetamido-2,6-dideoxy-a-d-*xylo*-4-hexulose, which is followed by the formation of UDP-4-amino-4,6-dideoxy-α-d-GlcNAc by PglE. Then, PglD transfers the acetyl group. PglC links UDP-diNAcBac to a lipid-linked precursor, undecaprenyl phosphate (Und-P). PglA and PglJ catalyze sequential reactions to add α-1,3- and α-1,4-linked N-acetylgalactosamine. Then, PglH adds three α-1,4-linked GalNAc. In the final step, PglI attaches β-1,3-linked glucose and completes the synthesis. The lipid-linked oligosaccharide is then flipped from the cytoplasmic side to the periplasmic side of the cell by PglK, an ABC transporter enzyme. Then, PglB, the oligosaccharyltransferase, transfers the heptasaccharide onto the glycosylation sequon (D/E-X_1_-N-X_2_-(S/T), where X_1_ and X_2_ cannot be proline).^17^ The discovery of the glycosylation machinery of *Campylobacter jejuni* (*C. jejuni*)^18^ and its recapitulation in laboratory workhorse *Escherichia coli* have greatly leveraged the possibilities in glycoengineering.^14^

The glycoengineering approach has been widely used in many different organisms to develop better therapeutics. Recombinant glycosylation can be used to alter biophysical and pharmacokinetic properties. One study showed that the addition of glycosylation sites to drug molecules leads to increased circulation times and binding abilities.^4^ Another study reported that glycosylation of a single-chain antibody improved the solubility and proteolytic stability.^19^ Glycoengineering was also employed to develop vaccines. Utilization of recombinant glycosylation offers an easy and cost-effective alternative to the vaccine production procedure where adjuvant and carrier proteins have to be covalently linked.^1^ Lastly, to produce glycomaterials with enhanced properties, biofilm proteins were N-linked glycosylated. In this study, it has been shown that the addition of N-glycans to biofilm proteins leads to a protein-based biomaterial with enhanced adsorption properties.^20^ In this study, we employed the N-linked glycosylation mechanism of *C. jejuni* to recombinantly produce glycosylated enzymes in *E. coli*. As a proof of concept, we chose the alkaline phosphatase (ALP) enzyme to investigate the effects of N-linked glycosylation on the behavior of the enzyme. We also performed secondary structure analysis under different conditions and assessed the enzymatic activity differences upon glycosylation.

## Materials and Methods

### Plasmid Construction and Bacterial Growth Conditions

All plasmids and primers were designed in silico using Benchling. To start with, constructs carrying the *phoA* gene encoding the *E. coli* ALP enzyme were fused in silico with a His-tag at the C-terminus for detection and purification purposes. Then, the glycosylation tag, DQNAT, was fused at different locations, and an ALP-6XHis-DQNAT was located under T7-lacO promoter in the pET22b plasmid. The glycosylation tag, DQNAT, was designed to be added by utilizing primer overhangs containing the “GATCAGAACGCGACC” sequence.

To acquire the plasmids, PCR reactions were performed using Q5 High-Fidelity DNA Polymerase (New England Biolabs Inc., Boston, USA) by following the manufacturer’s instructions. Primers that were used in PCR reactions were ordered from Oligomer. Annealing temperatures for PCR reactions were calculated by using the NEB Tm Calculator tool.

The amplicons were extracted from agarose gel using a gel extraction kit (M&N, REF 740609.50) according to the manufacturer’s instructions. Then, both pET22b and ALP fragments were digested using XbaI and XhoI restriction enzymes. DNA fragments were again recovered from agarose gel, and the backbone and ALP fragments were joined together using T4 DNA ligase.

The *phoA*-DQNAT pET22b plasmid was digested with *Bam*HI to insert the *pglB* gene. Amplification and insertion of the *pglB* gene encoding the pglB enzyme in the pgl pathway were performed via Gibson assembly. The gene was amplified using the primers:

- 5′CTAGAATTAAAGAGGAGAAAGGTACGCGGATCCATGTTGAAAAAAGAGTATTTAAAAA3′ and
- 5′CCAGTGCAATAGTGCTTTGTTTCATGTTTCTCCTCTTTAATACTAGTTTAAATTTTAAGTTTAAAAACCTTAG3′.

PCR products were mixed with 6× purple loading dye (New England Biolabs Inc. Boston, USA, B7024S) and run in 1% agarose gel. 1kb+ Ladder (New England Biolabs Inc. Boston, USA, N3200L) was utilized to track the DNA length. To prepare agarose gels, 0.6 g of agarose was dissolved in 60 mL of 1× TAE (Tris base, acetic acid, and EDTA) buffer. Electrophoresis was performed for 30 min at 140 V. Bands confirmed by electrophoresis were extracted using the Macherey-Nagel GmbH & Co. (Düren, Germany) gel extraction and PCR cleanup kit, following the manufacturer’s instructions, and the yield was measured using a NanoDrop 2000 spectrophotometer (Thermo Fisher, Waltham USA, ND2000).

For restriction digestion reactions, New England Biolabs enzymes were used, and manufacturer’s instructions were followed. Fragments were run on an agarose gel and extracted as mentioned above. PCR parts were joined together using the Gibson assembly method. Gibson assembly products were transformed into chemically competent Dh5α cells. To perform the transformation, chemically competent cells were thawed on ice. Then, cloning products were added to cells and incubated for 20 min on ice. Heat shock was applied at 42 °C for 30 s, and cells were transferred to ice again for 2 min. One mL of LB medium was added, and cells were incubated at 37 °C for 1 h. After incubation, centrifugation was performed at 8000 G for 5 min, and the supernatant was discarded. The pellet was resuspended in residual supernatant and laid on agar plates with appropriate antibiotics. Agar plates were incubated at 37 °C O/N. After incubation, colonies were screened with colony PCR. Positive colonies were inoculated in LB medium with appropriate antibiotics. Plasmids were isolated using a Gene-JET Miniprep Kit (Thermo Scientific, Waltham USA, K0503). Isolated plasmids were verified by Sanger sequencing. Details of the amino-acid sequences of the cloned protein can be found in the Supporting Information.

### SDS-PAGE, Coomassie Blue Staining, and Immunoblotting

SDS-PAGE was performed both to verify the purity of the proteins and to observe the effects of proteolytic cleavage. For the verification part, 20 μL of 2 μg/mL protein in 25 mM Tris–HCl (Sigma-Aldrich, St. Louis, MO USA L2650) was mixed with a 6× SDS loading dye (375 mM Tris–HCl (pH 6.8), 9% (w/v) SDS, 50% (v/v) glycerol, 0.03% (v/v) bromophenol) and denatured at 95 °C for 5 min. SDS gel was prepared by the BioRad (Hercules, CA, USA) SDS Gel Casting System, and 20 μL of the mixture was loaded onto the gel. The proteins were run on the gel for nearly 2 h by applying 120–150 V. For observation of the effects of proteolytic cleavage, 20 μL of 100 μg/mL protein in Proteinase K (PrK), 20 μL of the purified protein in urea, and 20 μL of the purified protein in sodium phosphate buffer were mixed with the 6× SDS loading dye, and the same procedures were applied for the electrophoresis part. Coomassie blue staining was performed, and the gel was visualized by Image Lab Software (Bio-Rad, Hercules CA USA).

For whole-cell western and lectin blots, different concentrations of cells carrying the construct for ALP production were utilized to verify the presence of recombinant proteins and their glycosylated forms by applying the above-mentioned SDS-PAGE procedures. The proteins were then transferred to a polyvinylidene difluoride (PVDF) membrane (Thermo Fisher Scientific 88518, Waltham USA, K0503). The PVDF membrane was activated by methanol and then put in Turbo transfer buffer (Bio-Rad, Hercules CA USA). The Transblot Turbo Transfer System (Bio-Rad, Hercules CA USA) was used for semidry transferring in 7 min.

For western blotting, 1× Tris buffer saline with 0.1% of Tween (TBS-T) containing 5% skim milk was used as the blocking solution. The membrane was blocked with the blocking solutions for 2 h at room temperature. The primary antibody (Anti-His Mouse PTGLAB (Romemont, IL, 66005)) was diluted at 1:10,000 in the blocking solution, and the incubation step was done for 1 h at room temperature. The membrane was washed with 1× TBS-T (0.1%) three times for 5, 10, and 10 min. Secondary antibody (horseradish peroxidase-conjugated goat antimouse) (Abcam ab6789-1 MG) incubation was performed for 2 h at room temperature by diluting it at 1:10,000 in blocking solution. Finally, the wash steps were repeated. Visualization of the proteins on the membrane was performed by enhanced chemiluminescence (ECL) (Bio-Rad 170-5060, Hercules, CA USA) via Image Lab Software (Bio-Rad).

For lectin blotting, the blocking solution was filtered with 1× TBS-T (0.05%) containing 3% bovine serum albumin. The membrane was blocked with the blocking solution for 1 h at room temperature. Soybean agglutinin (SBA) antibody (Sigma-Aldrich, St. Louis, MO USA L2650) was diluted at a 1:5000 ratio in the blocking solution, and its incubation was done for 2 h at room temperature. Wash steps were performed with 1× TBS-T (0.05%) five times for 5 min each. Visualization steps were also performed by ECL. Details of western blotting along with results can be found in the Supporting Information.

### Recombinant Protein Expression and Extraction

BL21 (DE3) cells transformed with plasmids were inoculated from the stock in ZMY052 autoinduction media (tryptone, yeast extract, Na_2_HPO_4_, KH_2_PO_4_, NH_4_Cl, Na_2_SO_4_, and MgSO_4_·7H_2_O, Sigma-Aldrich, St. Louis, MO USA) prepared according to Studier et al.^21^ After a 20–24 h of incubation, protein extraction steps were performed. Cells precipitated by centrifuging at 3500 G for nearly 30 min were resuspended with 10 mM imidazole buffer (20 mM sodium phosphate, 0.5 M NaCl, 10 mM imidazole, pH 7.4, Sigma-Aldrich, St. Louis, MO USA). Proteases were inhibited with the addition of 1 mM phenylmethanesulfonyl fluoride (AMRESCO Inc., Cleveland OH USA), and cells were lysed by sonication. In the sonication step, 30 s pulse-on and 60 s pulse-off cycles with an amplitude of 35% were applied 5 times. To collect recombinant proteins, lysed cells were centrifuged at 12,000 rpm for 1 h, and total protein was extracted as the supernatant and stored at +4 °C.

### Recombinant Protein Purification by Cobalt Resin

For the purification of recombinant proteins, 10 mM imidazole in 20 mM sodium phosphate and 0.5 M NaCl were used as the binding buffer. The lysis supernatant containing total protein was added to the equilibrated cobalt resin (Thermo Fisher, 89964, Waltham USA, K0503), and the mixture was rotated for 1 h at room temperature to provide the binding of His-tagged ALP to the resin. Unbound proteins were discarded, and the resin was washed three times with 10 mM imidazole binding buffer. Bound proteins were eluted three times with 150 mM imidazole elution buffer (20 mM sodium phosphate and 0.5 M NaCl). Proteins in the elution buffer were exchanged with 25 mM Tris–HCl (pH 8), sodium phosphate buffer (1 M Na_2_HPO_4_ and 1 M NaH_2_PO_4_), and 8 M urea (Sigma-Aldrich, 51457) via a HiTrap Desalting column (Sigma-Aldrich, GE17140801) for further experiments. Quantification of the recombinant proteins was performed by the Pierce BCA assay kit (Thermo Fisher Scientific 23225, Waltham USA, K0503) according to the manufacturer’s instructions.

### Glycosylation Rate Estimation

The extent of glycosylation of the constructs was determined using western blotting analysis. The analysis of bands was conducted using Vilber Evolution Capt Edge software after viewing the membrane with the Vilber Lourmat FUSION SOLO 6 (Collégien, France) imaging equipment. In the software, the quantification application was selected. Background signals were subtracted from the image, and equal areas were selected for the most precise calculation.

### ALP Activity Assay

Enzymatic activity was determined for both the wild type and the glycosylated form of ALP by measuring the catalysis of *para*-Nitrophenylphosphate (pNPP) (Sigma-Aldrich, St. Louis, MO USA L2650), one of the substrates of the ALP enzyme that is cleaved and converted into a colored product by the ALP enzyme. The pNPP reaction buffer (0.1 M glycine, 1 mM MgCl_2_, and 1 mM ZnCl_2_; Sigma-Aldrich, St. Louis, MO USA) was used to make pNPP substrate solutions. Incubations were performed on a thermal cycler (Bio-Rad, Hercules CA USA). 50 μL aliquot of proteins diluted to 2 μg/mL with 25 mM Tris–HCl (pH 8) was mixed with 50 μL of pNPP substrates in this part of the experiment. The absorbance of the wells at 405 nm was measured at 37 °C for 20 min. The Michaelis–Menten curve was created using various pNPP concentrations (0, 1, 2, 3, 4, and 5 mM). The reaction rate was estimated by using the pNPP standard curve.

The stability and activity variations of these enzymes were determined by calculating and comparing their enzymatic activities under various conditions. For this purpose, proteins were incubated at 55, 75, and 95 °C for 15, 30, 60, and 120 min for each temperature. Then, the enzymatic activities of the incubated proteins were measured as previously explained to observe the effects of increase in temperature and incubation times.

Effects of pH on enzymatic activities of recombinant proteins were calculated by measuring the catalysis of the pNPP substrate. Therefore, proteins were diluted to 2 μg/mL with 25 mM Tris–HCl having pH = 5, 6, 7, 8, 9, 10, and 11. In this stage of the experiment, 50 μL of proteins was combined with 50 μL of pNPP substrates, and enzyme activities were measured as previously explained.

Treatments with PrK and urea were applied to test the stabilities of the enzymes. Thus, PrK was applied for varying incubation times (60, 120, and 240 min) as a final concentration of 0.5 μM. Then, different concentrations of PrK (Sigma-Aldrich, St. Louis, MO USA) were also applied (e.g., final concentrations of 10, 1, 0.5, 0.05, and 0.005 μM), as time is kept constant as 60 min. Additionally, activities of 50 μL of 2 μg/mL protein were measured in different urea concentrations (8, 1, 0.1, 0.01, and 0.001 M) after an incubation process lasting for 1 h at 37 °C.

### Circular Dichroism

Circular dichroism (CD, JASCO J-815, Tokyo, Japan) studies were carried out to observe the changes in secondary structures of recombinant proteins with changes in the temperature, pH, and the presence of proteinase or a denaturant. 1 μM of each protein in Tris buffer (pH 8.0) was used for the experiments. For pH, a sodium phosphate buffer was used. CD, voltage, and absorbance were chosen as the three channels. The wavelength was set to 190–250 nm, with 4 s digital integration time (D.I.T.), 1 nm bandwidth, wavelength range, standard sensitivity, 100 nm/min scanning speed, and 3 repeat accumulation modes.

### Data Representation and Statistical Analysis

Data represented in the figures were drawn using GraphPad Prism 9. Statistical analysis was performed using the software tools. To determine significance levels indicated in the figures, calculations were done using 2-way ANOVA which performed multiple comparisons between the same time or concentration values of ALP-DQNAT and Glycosylated ALP-DQNAT. Levels of significance were reported in GraphPad Prism as *P*-values, meaning *P* <0.0001 is “****”, *P* <0.0002 is “***”, *P* <0.0021 is “**”, *P* <0.0332 is “*”, and *P* >0.1234 is “non-significant” (NS). All assays were performed in triplicate, and error bars in graphs represent the standard deviation of samples. Visual representations were created with Biorender.com.

## Results

### Expression of ALP and Glycosylated ALP in *E. coli*

**Figure 1:**
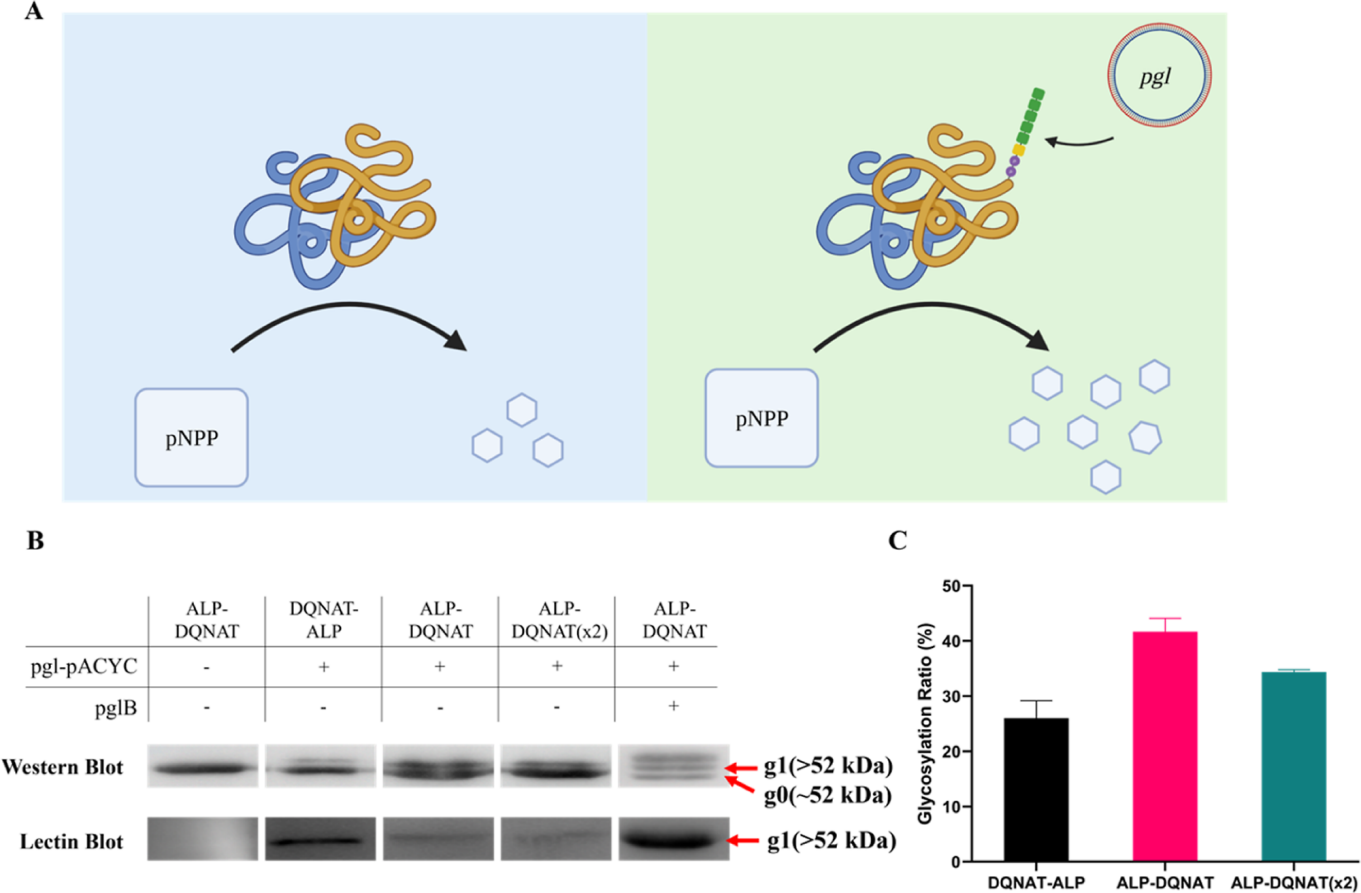
(A) Illustration of the effect of recombinant glycosylation on the ALP enzyme. (B) Western and lectin blot results of the designed constructs. Unglycosylated ALP is denoted as “g0” and glycosylated ALP is denoted as “g1”. (C) Calculated glycosylation rate of the constructs based on the western blot results.

In this study, we focused on the effect of *N-linked glycosylation* of *C. jejuni* on the ALP enzyme’s behavior. Although ALP is widely studied for decades, recombinant glycosylation of an enzyme and the effect of glycosylation have not been investigated before. By making an analogy to eukaryotic glycosylation, it is anticipated that recombinantly glycosylated enzymes will perform better at certain conditions compared to the native enzyme (Figure 1A). Given that the success of the glycosylation event depends on the accessibility of the recognition motif by PglB, two constructs were designed that carry glycosylation motifs at the N-terminus (DQNAT-ALP) and the C-terminus (ALP-DQNAT). Also, another construct with two recognition motifs at the C-terminus (ALP-DQNAT(x2)) was designed. An illustration of the constructs can be seen in Figure S1. Designed constructs were cotransformed into BL21 (DE3) cells with pgl-pACYC plasmid as well as without pgl-pACYC as a control. Western blot results yielded two different bands, suggesting there are two states of ALP. SBA lectin blot (Figure 1B) analysis confirms that the second states were glycosylated. Glycosylation rates were estimated by calculating band intensities within the lanes in western blot (Figure 1C). According to the results, ALP-DQNAT, where the glycosylation tag is at the C-terminus of the ALP enzyme, was estimated to be the highest glycosylated construct, with 45% glycosylation. Moreover, repeating the recognition motif did not increase the glycosylation extent further. On the contrary, a slight decrease was observed.

### Improving the Glycosylation Efficiency in the Cell

The N-linked glycosylation machinery of *C. jejuni* adds glycan groups to target proteins in the periplasm of the cell. To eliminate unglycosylated ALP in the cytoplasm, periplasmic protein extraction by osmotic shock has been performed. Western blot analysis indicated that the glycosylation extent does not change significantly (Figure S8), suggesting that most of the ALP that is produced successfully translocates to the periplasm. Therefore, to increase the glycosylation efficiency in the periplasm, we overexpressed the PglB enzyme in the cell. Western blot results yielded an extra band (Figure 1B, lane 5), suggesting three states. Lectin blot results contradicted the western blot ones and indicated that there is only one glycosylated state. Therefore, the extra band most likely does not correspond to *N-glycosylated* ALP. However, during His-tag purification, the extra band was eliminated during the purification process. The eluate was checked with western blot and lectin blot to confirm the glycosylation and with SDS-PAGE for purity (Figure S9). After the protein is purified, approximately a 52% glycosylated batch of ALP was obtained, and it will be referred to as “Glycosylated ALP-DQNAT” from now on.

### Assessing the Enzymatic Activity of Glycosylated ALP

After successful glycosylation of ALP and achieving increased glycosylation rate, we investigated the effect of recombinant glycosylation on the enzyme’s behavior. pNPP is the substrate of ALP enzyme, and after it is cleaved by ALP, it forms a yellow-colored product. The ALP activity can be assessed by the rate of color change in a transparent medium.^22^ There are different approaches to compare enzymes that catalyze the same reaction (e.g., ALP-DQNAT and Glycosylated ALP-DQNAT). Michaelis–Menten constant has been vastly utilized to assess the performance of enzymes over the last century.^23^ While it is useful to evaluate enzymes in their natural environments, it does not take into account industrial processes, such as a high substrate concentration, nonphysiological pH, and high temperature.^24^ Furthermore, with the advances in genetic engineering, such as directed evolution, comparing enzyme variants acting on the same substrate becomes tricky utilizing *k*_cat_/*K*_M_.^25^ Therefore, new evaluation methods and parameters are required. Since the important parameter is the completion of the reaction in most of the industrial processes, tracking product formation and comparing enzymes accordingly would be more suited.^26^

**Figure 2:**
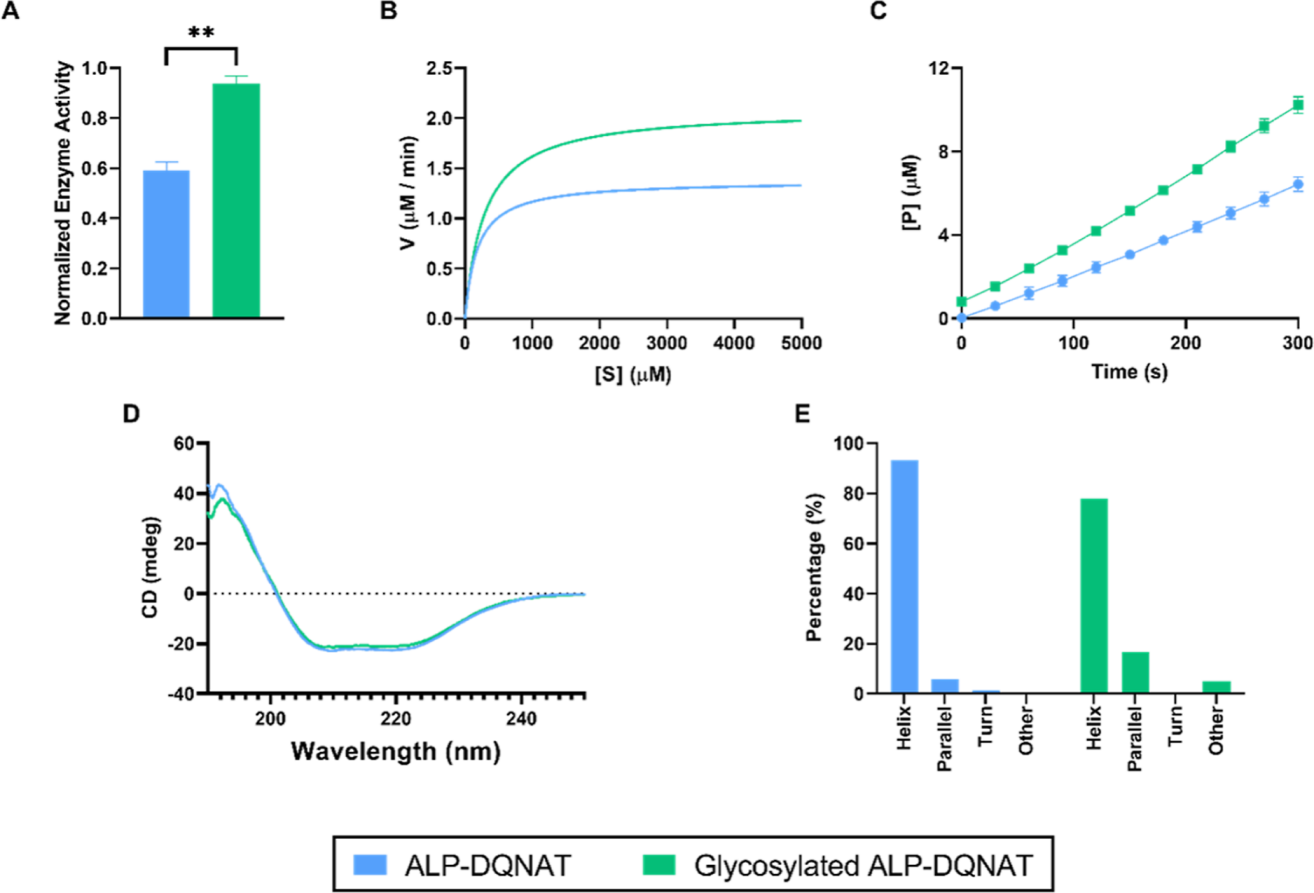
Analysis of ALP-DQNAT and Glycosylated ALP-DQNAT under optimal conditions. (A) Enzyme activity of ALP-DQNAT and Glycosylated ALP-DQNAT. (B) Michaelis–Menten analysis of ALP-DQNAT and Glycosylated ALP-DQNAT. (C) Plot of substrate conversion performed by ALP-DQNAT and Glycosylated ALP-DQNAT over time. (D) CD spectral results of ALP-DQNAT and Glycosylated ALP-DQNAT. (E) Secondary structure predictions of ALP-DQNAT and Glycosylated ALP-DQNAT using CD data.

We performed enzyme activity assays to assess the effect of glycosylation under optimal conditions. The results showed that Glycosylated ALP-DQNAT performed 1.5-fold better than the native enzyme (Figure 2A). We also performed a Michaelis–Menten analysis to evaluate the differences in enzyme kinetics (Figure 2B). The analysis showed that ν_max_ is 1.0376 ∓ 0.068 and 2.088 ∓ 0.060 μM/min for ALP-DQNAT and Glycosylated ALP-DQNAT, respectively. The *K*_m_ value was also increased from 180 ∓ 110.1 to 290 ∓ 70.47 μM upon glycosylation. Furthermore, to show the substrate conversion into the product over time and calculate the average velocity, a product formation versus time graph was drawn (Figure 2C). The average velocities and *k*_cat_/*K*_m_ values for ALP-DQNAT and Glycosylated ALP-DQNAT were calculated as 0.959 ∓ 0.17 and 1.448 ∓ 0.15 μM s^–1^ and 6.69 × 10^–3^ and 6.31 × 10^–3^ μM^–1^ s^1^, respectively.

To understand the changes in the activity of the enzyme, changes in the secondary structures upon glycosylation were also analyzed. CD is a widely utilized method to study the secondary structures of proteins.^27^ To determine the secondary structures, a wavelength range between 190 and 250 nm was utilized. Results showed that N-linked glycosylation did not affect the CD spectra significantly and ALP (Figure 2D). Similar results have been reported previously.^20,28^ To predict the secondary structures, the BestSel online tool was utilized.^29^ Results indicated that ALP preserved its α-helical structure (Figure 2E).

### N-Linked Glycosylation Increases the Stability of ALP Enzyme

**Figure 3:**
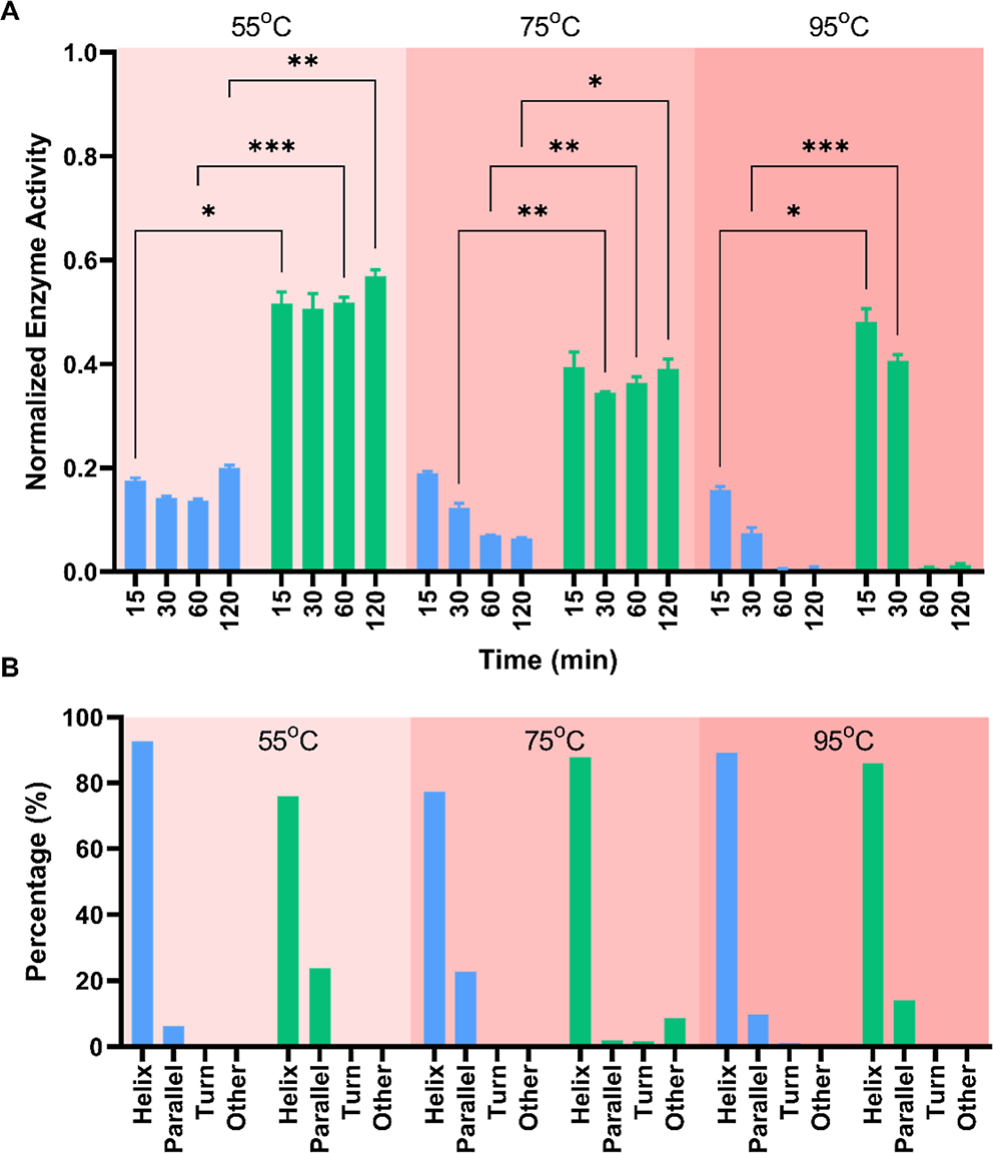
Glycosylation leads to a better-performing ALP. (A) Enzyme activity results of ALP-DQNAT and Glycosylated ALP-DQNAT incubated at elevated temperatures. (B) Secondary structure predictions of ALP-DQNAT and Glycosylated ALP-DQNAT.

To test the effect of glycosylation on the enzyme activity at elevated temperatures, both enzymes were incubated at 55, 75, and 95 °C for varying incubation times (15, 30, 60, and 120 min) before enzyme activity assays (Figure 3A). After incubation at 55 °C, the activity of both enzymes decreased compared to that of the untreated groups in Figure 2A. However, Glycosylated ALP-DQNAT preserved 55% of its activity, whereas ALP-DQNAT showed 29% of its activity under optimal conditions. At 75 °C, ALP-DQNAT enzyme becomes susceptible to incubation time as well and continues losing its activity as the incubation time increases. Interestingly, Glycosylated ALP-DQNAT preserved its activity with increasing incubation times. Therefore, the fold change between ALP-DQNAT and Glycosylated ALP-DQNAT was 2-fold when treated for 15 min, and it increased to 6.1-fold after 120 min treatment. At 95 °C, both enzymes cease to work after 30 min of incubation. Overall, the results indicated that Glycosylated ALP-DQNAT performed better under all conditions and preserved more of its activity at elevated temperatures.

We also investigated the effect of increased temperatures on the secondary structure of the enzymes. Both enzymes were treated with elevated temperatures (55, 75, and 95 °C) for 2 h. Measurements were taken immediately after treatment. CD results were used as input for BeStSel tool, and the secondary structure predictions are shown in Figure 3B. According to the results, 55 and 95 °C treatments did not cause a significant change in the secondary structure. However, at 75 °C, a parallel beta-sheet structure of glycosylated ALP was diminished, and it was compensated by alpha helices and other secondary structures. This change in the secondary structure may explain the reason why ALP-DQNAT activity was weakened over time, whereas Glycosylated ALP-DQNAT protected its activity.

### Glycosylated ALP-DQNAT Is More Active at Suboptimal pH Conditions and More Alkaline pH

**Figure 4:**
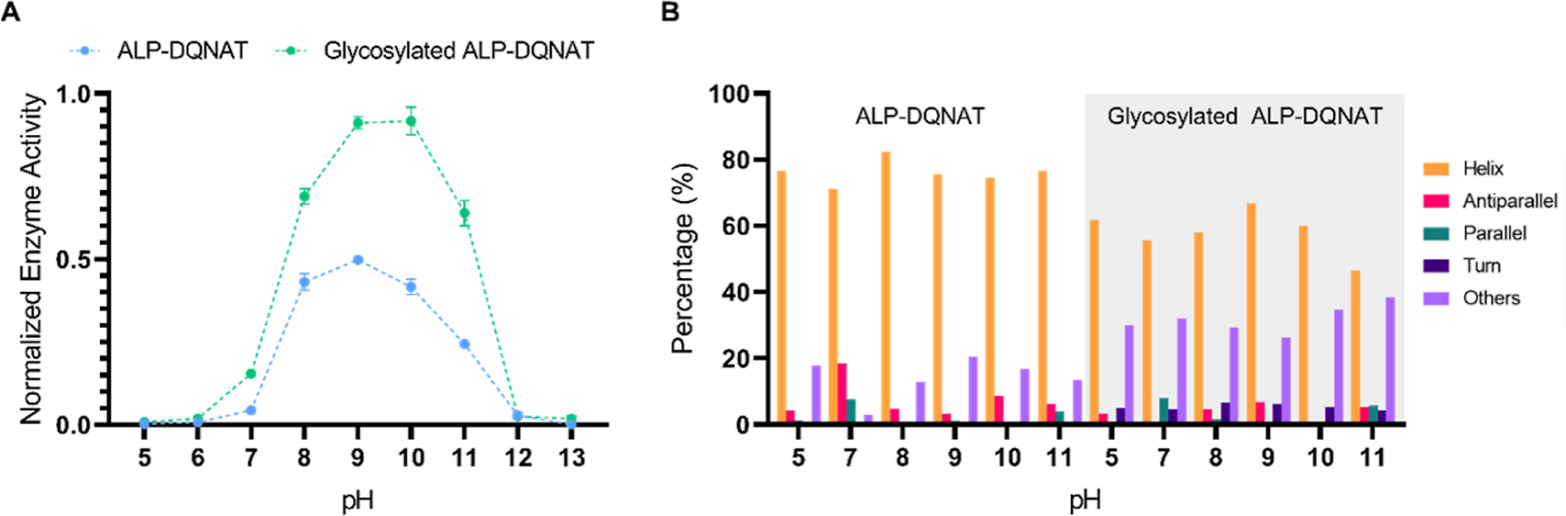
Working pH range screening for ALP-DQNAT and Glycosylated ALP-DQNAT. (A) Glycosylated ALP-DQNAT performs better for all pH conditions tested. (B) Secondary structure predictions of ALP-DQNAT and Glycosylated ALP-DQNAT. We observed a slight shift to more alkaline pH conditions upon glycosylation.

pH is one of the most significant factors influencing the enzyme activity. Therefore, pH conditions should be well arranged for enzymes to work efficiently. *E. coli* ALP enzyme has an optimum pH of 8.0.^30^ To examine how glycosylation affects the working pH range of the enzyme, we performed enzyme activity assays under different pH conditions, ranging from pH 5.0 to 13.0 (Figure 4A). At pH 5.0, 6.0, 12.0, and 13.0, no enzyme activity was detected. At other tested pH conditions, Glycosylated ALP-DQNAT activity was significantly higher, compared to that of ALP-DQNAT. Furthermore, it can be said that, upon glycosylation, the optimum pH for the enzyme has shifted to more alkaline conditions, as the highest activity was seen at pH 10.0. This is in good agreement with other studies that examine the effect of glycosylation.^31,32^

We also studied secondary structures at different pH conditions, where enzyme activity was observed (Figure 4B). However, no significant difference that may account for varying enzyme activity results was observed.

### Glycosylation Protects ALP against Proteolytic Cleavage

Another important characteristic of proteins is their stability against proteolytic cleavage. Proteases are enzymes that degrade proteins into smaller peptides. N-Linked glycosylation was stated to contribute to stability against proteases.^1,32^ Glycosylation provides this protection by shielding protein regions from proteases.^33^ We utilized a serine protease, PrK, to assess the stability of enzymes against proteases. PrK also has an optimum pH of 8.0; therefore, it is convenient to use in the same medium.

**Figure 5:**
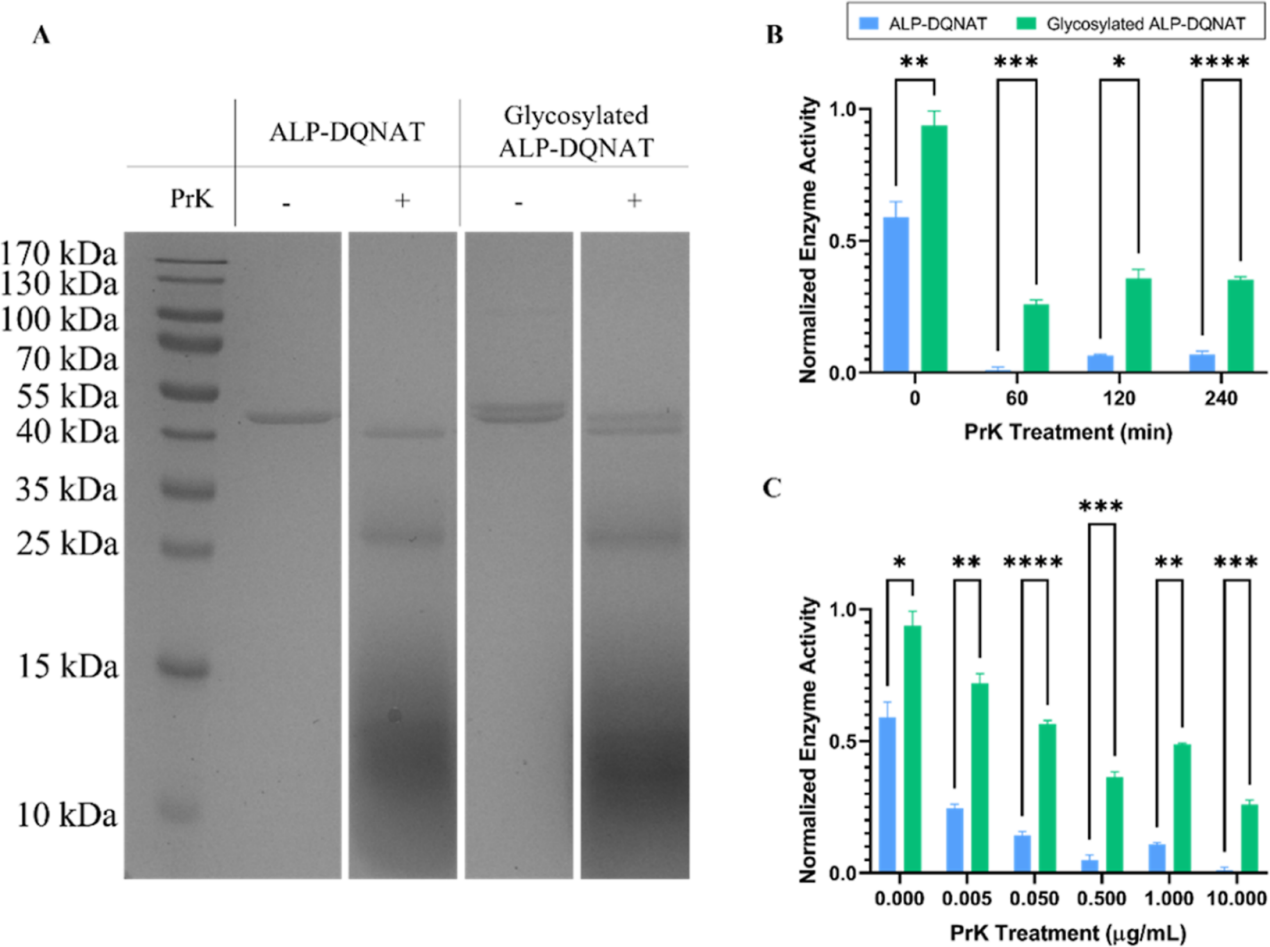
ALP-DQNAT and Glycosylated ALP-DQNAT are assessed in terms of their stability against proteolytic cleavage. (A) SDS-PAGE analysis was performed to observe proteolytic cleavage performed by PrK. (B) ALP-DQNAT and Glycosylated ALP-DQNAT were treated with PrK for varying incubation times. (C) Increasing PrK was applied to both enzymes to investigate the alterations in the enzymatic activity.

We first checked proteolytic cleavage on SDS gel (Figure 5A). SDS gel confirms that both enzymes were subject to degradation. It was observed that both proteins were not able to protect their full size. We also investigated the effect of incubation time with PrK (Figure 5B). It was seen that 60 min of treatment is sufficient to observe the effects of PrK. Therefore, the incubation time was selected as 60 min for concentration-dependent analysis. Next, we applied increasing concentrations of PrK (Figure 5C). It was observed that the activity fold change increases as the PrK concentration increases. At 10 μg/mL, the fold change increases 24-fold, and ALP activity is very limited.

### Enzyme Activity Varies under Denaturing Conditions

**Figure 6:**
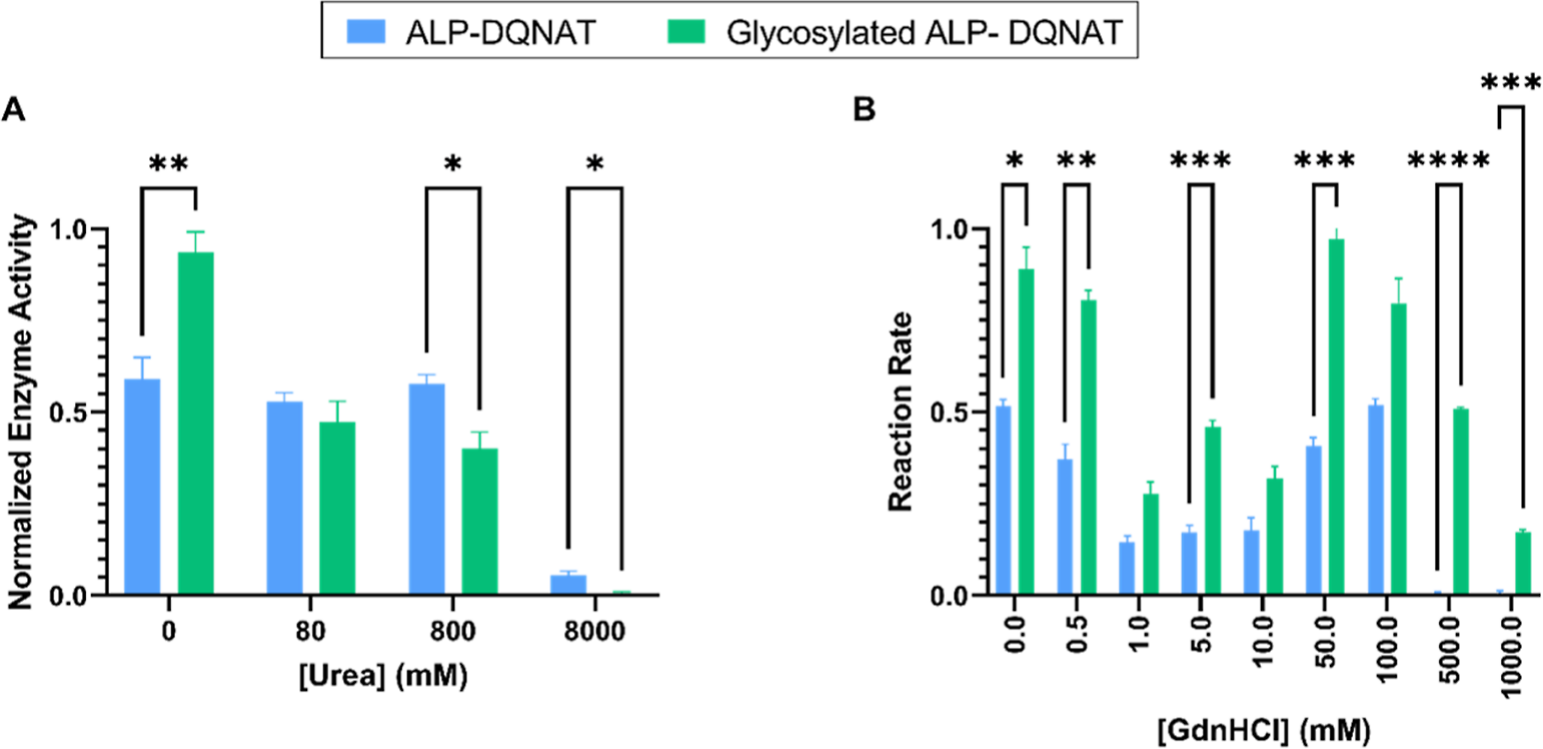
Different denaturing conditions have different consequences for enzyme activity upon glycosylation. (A) Enzyme activity in the presence of urea. Urea has a diminishing effect on the activity of Glycosylated ALP-DQNAT(*P*-value = NS). (B) Enzyme activity in the presence of Gdn-HCl. Glycosylated ALP-DQNAT performs better under all conditions tested with *P*-value = 0.0003.

We investigated the enzymatic behaviors of both ALP-DQNAT and Glycosylated ALP-DQNAT under denaturation conditions. We utilized two common denaturing agents, guanidine hydrochloride and urea. We first started examining the effects of urea on the enzyme activity (Figure 6A). Contrary to previous results, we observed that in urea the enzyme activity of ALP-DQNAT is higher under all conditions tested. Then, we also examined enzyme activities in guanidinium hydrochloride (Gdn-HCl) (Figure 6B). Enzymes performed as expected with the addition of Gdn-HCl. It has been seen that Glycosylated ALP-DQNAT performed better at all Gdn-HCl concentrations and continued working under conditions where ALP-DQNAT activity was lost. A surprising result was observed at moderate concentrations. Moderate concentrations of Gdn-HCl lead to an unexpected stimulation of both enzymes’ activities. This effect of Gdn on ALP has been previously found and discussed and therefore results are in agreement with the literature.^34^ Therefore, glycosylation enhanced the stability of ALP-DQNAT against Gdn-HCl.

## Discussion

In this work, we investigated how recombinant glycosylation affects the activity and structure of an enzyme (e.g., ALP). First, to achieve high glycosylation rates, different strategies were followed. When the *pglB* gene encoding for the key oligosaccharyltransferase enzyme in the pathway was overexpressed, an extra band emerged in western blot, which cannot be confirmed as a glycosylated state by lectin blot. The simplest explanation could be that ALP-DQNAT is diglycosylated at a different site. To explore this possibility, we used an in silico tool GlycoPP to uncover putative N-linked glycosylated sites.^35^ GlycoPP showed that three more sites are potentially N-linked glycosylated (Table S1). Similar results were reported in the literature before.^36^ An alternative possibility is that another immature glycan is attached to ALP-DQNAT upon PglB overexpression. It has been previously reported by many studies that PglB has relaxed specificity.^37,38^ Although it is known that *C. jejuni* utilizes only one type of oligosaccharide, immature glycans could be attached to one or more of the potential glycosylated sites. In this way, lectin blot could not detect the immature glycans; therefore, a slower migrating band was observed only in western blot. In the same manner, O antigens could be attached to ALP-DQNAT as well, and they also could escape lectin blot, resulting in the same situation, since the strain utilized already synthesized O antigens.^38^

Engineering of enzymes to obtain durable variants has always been of great interest in the bioindustry in terms of thermostability, stability at a wider pH range, and stability against proteases and denaturants. When both ALP-DQNAT and Glycosylated ALP-DQNAT were tested at elevated temperatures, the activity fold change increased, as the treatment time increased due to ALP-DQNAT activity being limited by the effect of temperature. On the other hand, Glycosylated ALP-DQNAT activity is maintained at comparable levels. It can be speculated that upon glycosylation, ALP becomes more durable at increased temperatures and maintains its catalytic activity without significant loss. Although it is well known that glycosylation, in general, may alter the stability of proteins and it was also shown at elevated temperatures,^33,39,40^ to date, there has been no evidence that *C. jejuni* N-linked glycosylation contributes to the thermostability of the enzymes.

When both enzymes were tested under denaturing conditions, we observed the reverse effect of glycosylation. The addition of urea reversed the fashion followed in the enzymatic activities and diminished the activity of glycosylated ALP more, compared to unglycosylated ALP. Later we found out that, N-glycans react with high concentrations of urea and lead to the formation of an artifact with the combination of urea and glycan, as described in Omtvedt et al.^41^ Therefore, we speculated that this may explain the decrease in enzyme activities.

Overall, the results presented above indicate that glycosylation of ALP-DQNAT added a significant value to the enzyme’s characteristics, which makes them better tailored for the realm of bioprocess conditions. We anticipate that N-linked glycosylation allows rapid enhancement of enzymes for industrial processes and offers improved features of enzymes in terms of temperature, pH, proteolytic stability, and denaturing conditions.

## Supporting Information Available

Illustrations of ALP-DQNAT constructs, plasmid maps and sequencing results, gel images for glycosylation rate estimation, protein purity analysis and lectin blots, in silico glycosylation site analysis, and *sequences of ALP constructs* (PDF)

## References

[ref1] Kightlinger W.; Warfel K. F.; DeLisa M. P.; Jewett M. C. Synthetic Glycobiology: Parts, Systems, and Applications. ACS Synth. Biol. 2020, 9 (7), 1534–1562. 10.1021/acssynbio.0c00210. 32526139 PMC7372563

[ref2] Dell A.; Galadari A.; Sastre F.; Hitchen P. Similarities and differences in the glycosylation mechanisms in prokaryotes and eukaryotes. Int. J. Microbiol. 2010, 2010, 148178 10.1155/2010/148178. 21490701 PMC3068309

[ref3] Baker J. L.; Celik E.; DeLisa M. P. Expanding the glycoengineering toolbox: the rise of bacterial N-linked protein glycosylation. Trends Biotechnol. 2013, 31 (5), 313–323. 10.1016/j.tibtech.2013.03.003. 23582719

[ref4] Elliott S.; Lorenzini T.; Asher S.; Aoki K.; Brankow D.; Buck L.; Busse L.; Chang D.; Fuller J.; Grant J.; et al. Enhancement of therapeutic protein in vivo activities through glycoengineering. Nat. Biotechnol. 2003, 21 (4), 414–421. 10.1038/nbt799. 12612588

[ref5] Li H.; Sethuraman N.; Stadheim T. A.; Zha D.; Prinz B.; Ballew N.; Bobrowicz P.; Choi B. K.; Cook W. J.; Cukan M.; et al. Optimization of humanized IgGs in glycoengineered Pichia pastoris. Nat. Biotechnol. 2006, 24 (2), 210–215. 10.1038/nbt1178. 16429149

[ref6] Puetz J.; Wurm F. M. Recombinant Proteins for Industrial versus Pharmaceutical Purposes: A Review of Process and Pricing. Processes 2019, 7 (8), 476 10.3390/pr7080476.

[ref7] Ma B.; Guan X.; Li Y.; Shang S.; Li J.; Tan Z. Protein Glycoengineering: An Approach for Improving Protein Properties. Front. Chem. 2020, 8, 622 10.3389/fchem.2020.00622. 32793559 PMC7390894

[ref8] Abu-Qarn M.; Eichler J.; Sharon N. Not just for Eukarya anymore: protein glycosylation in Bacteria and Archaea. Curr. Opin. Struct. Biol. 2008, 18 (5), 544–550. 10.1016/j.sbi.2008.06.010. 18694827

[ref9] Nothaft H.; Szymanski C. M. Protein glycosylation in bacteria: sweeter than ever. Nat. Rev. Microbiol. 2010, 8 (11), 765–778. 10.1038/nrmicro2383. 20948550

[ref10] Szymanski C. M.; Yao R.; Ewing C. P.; Trust T. J.; Guerry P. Evidence for a system of general protein glycosylation in Campylobacter jejuni. Mol. Microbiol. 1999, 32 (5), 1022–1030. 10.1046/j.1365-2958.1999.01415.x. 10361304

[ref11] Scott N. E.; Parker B. L.; Connolly A. M.; Paulech J.; Edwards A. V.; Crossett B.; Falconer L.; Kolarich D.; Djordjevic S. P.; Hojrup P.; et al. Simultaneous glycan-peptide characterization using hydrophilic interaction chromatography and parallel fragmentation by CID, higher energy collisional dissociation, and electron transfer dissociation MS applied to the N-linked glycoproteome of Campylobacter jejuni. Mol. Cell. Proteomics 2011, 10 (2), S1–S18. 10.1074/mcp.M000031-MCP201. PMC3033663 20360033

[ref12] Lu Q.; Li S.; Shao F. Sweet Talk: Protein Glycosylation in Bacterial Interaction With the Host. Trends Microbiol. 2015, 23 (10), 630–641. 10.1016/j.tim.2015.07.003. 26433695

[ref13] Alemka A.; Nothaft H.; Zheng J.; Szymanski C. M. N-glycosylation of Campylobacter jejuni surface proteins promotes bacterial fitness. Infect. Immun. 2013, 81 (5), 1674–1682. 10.1128/IAI.01370-12. 23460522 PMC3648013

[ref14] Wacker M.; Linton D.; Hitchen P. G.; Nita-Lazar M.; Haslam S. M.; North S. J.; Panico M.; Morris H. R.; Dell A.; Wren B. W.; Aebi M. N-linked glycosylation in Campylobacter jejuni and its functional transfer into E. coli. Science 2002, 298 (5599), 1790–1793. 10.1126/science.298.5599.1790. 12459590

[ref15] St Geme J. W. III; Falkow S.; Barenkamp S. J. High-molecular-weight proteins of nontypable Haemophilus influenzae mediate attachment to human epithelial cells. Proc. Natl. Acad. Sci. U.S.A. 1993, 90 (7), 2875–2879. 10.1073/pnas.90.7.2875. 8464902 PMC46199

[ref16] Schwarz F.; Fan Y. Y.; Schubert M.; Aebi M. Cytoplasmic N-glycosyltransferase of Actinobacillus pleuropneumoniae is an inverting enzyme and recognizes the NX(S/T) consensus sequence. J. Biol. Chem. 2011, 286 (40), 35267–35274. 10.1074/jbc.M111.277160. 21852240 PMC3186387

[ref17] Tan F. Y.; Tang C. M.; Exley R. M. Sugar coating: bacterial protein glycosylation and host-microbe interactions. Trends Biochem. Sci. 2015, 40 (7), 342–350. 10.1016/j.tibs.2015.03.016. 25936979

[ref18] Young N. M.; Brisson J. R.; Kelly J.; Watson D. C.; Tessier L.; Lanthier P. H.; Jarrell H. C.; Cadotte N.; St Michael F.; Aberg E.; Szymanski C. M. Structure of the N-linked glycan present on multiple glycoproteins in the gram-negative bacterium, Campylobacter jejuni. J. Biol. Chem. 2002, 277 (45), 42530–42539. 10.1074/jbc.M206114200. 12186869

[ref19] Lizak C.; Fan Y. Y.; Weber T. C.; Aebi M. N-Linked glycosylation of antibody fragments in Escherichia coli. Bioconjugate Chem. 2011, 22 (3), 488–496. 10.1021/bc100511k. 21319730

[ref20] Sahin Kehribar E.; Isilak M. E.; Bozkurt E. U.; Adamcik J.; Mezzenga R.; Seker U. O. S. Engineering of biofilms with a glycosylation circuit for biomaterial applications. Biomater. Sci. 2021, 9 (10), 3650–3661. 10.1039/D0BM02192J. 33710212

[ref21] Studier F. W. Protein production by auto-induction in high-density shaking cultures. Protein Expression Purif. 2005, 41 (1), 207–234. 10.1016/j.pep.2005.01.016. 15915565

[ref22] Sayler G. S.; Puziss M.; Silver M. Alkaline phosphatase assay for freshwater sediments: application to perturbed sediment systems. Appl. Environ. Microbiol. 1979, 38 (5), 922–927. 10.1128/aem.38.5.922-927.1979. 16345464 PMC243610

[ref23] Kim J. K.; Tyson J. J. Misuse of the Michaelis-Menten rate law for protein interaction networks and its remedy. PLoS Comput. Biol. 2020, 16 (10), e1008258 10.1371/journal.pcbi.1008258. 33090989 PMC7581366

[ref24] Carrillo N.; Ceccarelli E.; Roveri O. Usefulness of kinetic enzyme parameters in biotechnological practice. Biotechnol. Genet. Eng. Rev. 2010, 27, 367–382. 10.1080/02648725.2010.10648157. 21415905

[ref25] Eisenthal R.; Danson M. J.; Hough D. W. Catalytic efficiency and kcat/KM: a useful comparator?. Trends Biotechnol. 2007, 25 (6), 247–249. 10.1016/j.tibtech.2007.03.010. 17433847

[ref26] Fox R. J.; Clay M. D. Catalytic effectiveness, a measure of enzyme proficiency for industrial applications. Trends Biotechnol. 2009, 27 (3), 137–140. 10.1016/j.tibtech.2008.12.001. 19193465

[ref27] Greenfield N. J. Using circular dichroism spectra to estimate protein secondary structure. Nat. Protoc. 2006, 1 (6), 2876–2890. 10.1038/nprot.2006.202. 17406547 PMC2728378

[ref28] Wu C. Y.; Lin C. W.; Tsai T. I.; Lee C. D.; Chuang H. Y.; Chen J. B.; Tsai M. H.; Chen B. R.; Lo P. W.; Liu C. P.; et al. Influenza A surface glycosylation and vaccine design. Proc. Natl. Acad. Sci. U.S.A. 2017, 114 (2), 280–285. 10.1073/pnas.1617174114. 28028222 PMC5240703

[ref29] Micsonai A.; Wien F.; Bulyaki E.; Kun J.; Moussong E.; Lee Y. H.; Goto Y.; Refregiers M.; Kardos J. BeStSel: a web server for accurate protein secondary structure prediction and fold recognition from the circular dichroism spectra. Nucleic Acids Res. 2018, 46 (W1), W315–W322. 10.1093/nar/gky497. 29893907 PMC6031044

[ref30] Garen A.; Levinthal C. A fine-structure genetic and chemical study of the enzyme alkaline phosphatase of E. coli. I. Purification and characterization of alkaline phosphatase. Biochim. Biophys. Acta 1960, 38, 470–483. 10.1016/0006-3002(60)91282-8. 13826559

[ref31] Guo M. J.; Hang H. F.; Zhu T. C.; Zhuang Y. P.; Chu J.; Zhang S. L. Effect of glycosylation on biochemical characterization of recombinant phytase expressed in Pichia pastoris. Enzym. Microb. Technol. 2008, 42 (4), 340–345. 10.1016/j.enzmictec.2007.10.013.

[ref32] Dotsenko A. S.; Gusakov A. V.; Rozhkova A. M.; Sinitsyna O. A.; Nemashkalov V. A.; Sinitsyn A. P. Effect of N-linked glycosylation on the activity and other properties of recombinant endoglucanase IIa (Cel5A) from Penicillium verruculosum. Protein Eng. Des. Sel. 2016, 29 (11), 495–502. 10.1093/protein/gzw030. 27440076

[ref33] Sola R. J.; Griebenow K. Effects of glycosylation on the stability of protein pharmaceuticals. J. Pharm. Sci. 2009, 98 (4), 1223–1245. 10.1002/jps.21504. 18661536 PMC2649977

[ref34] Rao N. M.; Nagaraj R. Anomalous stimulation of Escherichia coli alkaline phosphatase activity in guanidinium chloride. Modulation of the rate-limiting step and negative cooperativity. J. Biol. Chem. 1991, 266 (8), 5018–5024. 10.1016/S0021-9258(19)67750-1. 2002044

[ref35] Chauhan J. S.; Bhat A. H.; Raghava G. P.; Rao A. GlycoPP: a webserver for prediction of N- and O-glycosites in prokaryotic protein sequences. PLoS One 2012, 7 (7), e40155 10.1371/journal.pone.0040155. 22808107 PMC3392279

[ref36] Ollis A. A.; Chai Y.; Natarajan A.; Perregaux E.; Jaroentomeechai T.; Guarino C.; Smith J.; Zhang S.; DeLisa M. P. Substitute sweeteners: diverse bacterial oligosaccharyltransferases with unique N-glycosylation site preferences. Sci. Rep. 2015, 5, 15237 10.1038/srep15237. 26482295 PMC4894442

[ref37] Wacker M.; Feldman M. F.; Callewaert N.; Kowarik M.; Clarke B. R.; Pohl N. L.; Hernandez M.; Vines E. D.; Valvano M. A.; Whitfield C.; Aebi M. Substrate specificity of bacterial oligosaccharyltransferase suggests a common transfer mechanism for the bacterial and eukaryotic systems. Proc. Natl. Acad. Sci. U.S.A. 2006, 103 (18), 7088–7093. 10.1073/pnas.0509207103. 16641107 PMC1459022

[ref38] Feldman M. F.; Wacker M.; Hernandez M.; Hitchen P. G.; Marolda C. L.; Kowarik M.; Morris H. R.; Dell A.; Valvano M. A.; Aebi M. Engineering N-linked protein glycosylation with diverse O antigen lipopolysaccharide structures in Escherichia coli. Proc. Natl. Acad. Sci. U.S.A. 2005, 102 (8), 3016–3021. 10.1073/pnas.0500044102. 15703289 PMC549450

[ref39] Khan R. H.; Rasheedi S.; Haq S. K. Effect of pH, temperature and alcohols on the stability of glycosylated and deglycosylated stem bromelain. J. Biosci. 2003, 28 (6), 709–714. 10.1007/BF02708431. 14660870

[ref40] Eriksen S. H.; Jensen B.; Olsen J. Effect of N-linked glycosylation on secretion, activity, and stability of alpha-amylase from Aspergillus oryzae. Curr. Microbiol. 1998, 37 (2), 117–122. 10.1007/s002849900348. 9662611

[ref41] Omtvedt L. A.; Royle L.; Husby G.; Sletten K.; Radcliffe C.; Dwek R. A.; Rudd P. M.; Harvey D. J. Artefacts formed by addition of urea to N-linked glycans released with peptide-N-glycosidase F for analysis by mass spectrometry. Rapid Commun. Mass Spectrom. 2004, 18 (19), 2357–2359. 10.1002/rcm.1632. 15384159

