# Supplementary material for: Glycosylation Circuit Enables Improved Catalytic Properties for Recombinant Alkaline Phosphatase
Source: ACS Omega. 2023 Aug 31;8(39):36218–27. doi: 10.1021/acsomega.3c04669 (PMC10552120; doi:10.1021/acsomega.3c04669)
Supplement: Supplementary file 1 — ao3c04669_si_001.pdf [file ao3c04669_si_001.pdf]

Supplementary information

A Glycosylation Circuit Enables Improved Catalytic Properties of Recombinant Alkaline Phosphatase

Eray Ulaş Bozkurt[1,†], İrem Niran Çağıl[1,‡], Ebru Şahin Kehribar[1], Musa Efe Işılak[1], Urartu Özgür Şafak Şeker*[1]

[1] UNAM- Institute of Materials Science and Nanotechnology,
National Nanotechnology Research Center, Bilkent University, Ankara, 06800 Turkey

Corresponding author:
Dr. Urartu Ozgur Safak Seker
urartu@bilkent.edu.tr

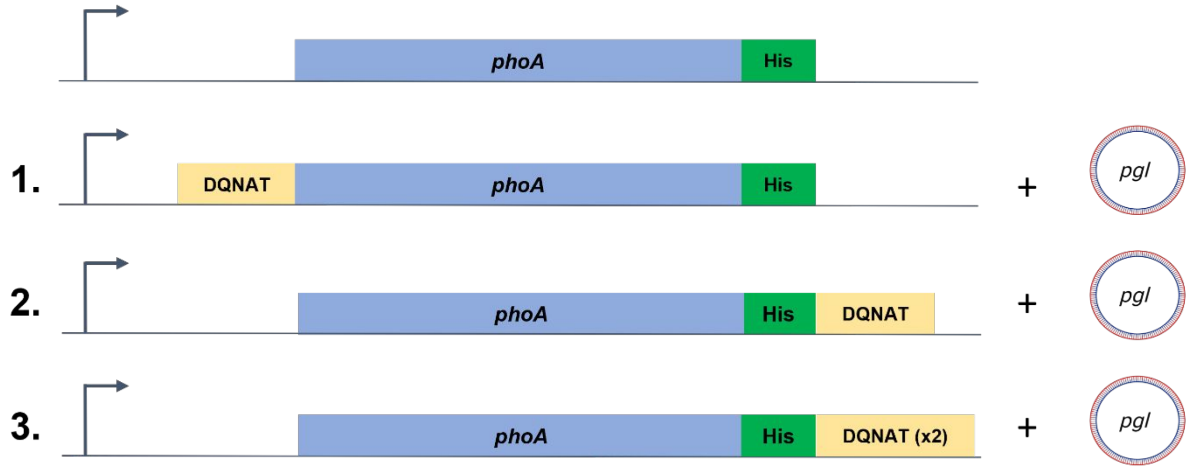

Figure S 1: The illustrations of designs utilized in this study.

† Current adress Technical University of Denmark, NNF Center for Biosustainability, Kemitorvet 220, Kongens Lyngby, 2800, Denmark

‡ Current address : LMU Munich – Ludwig-Maximilians-Universität München, Germany

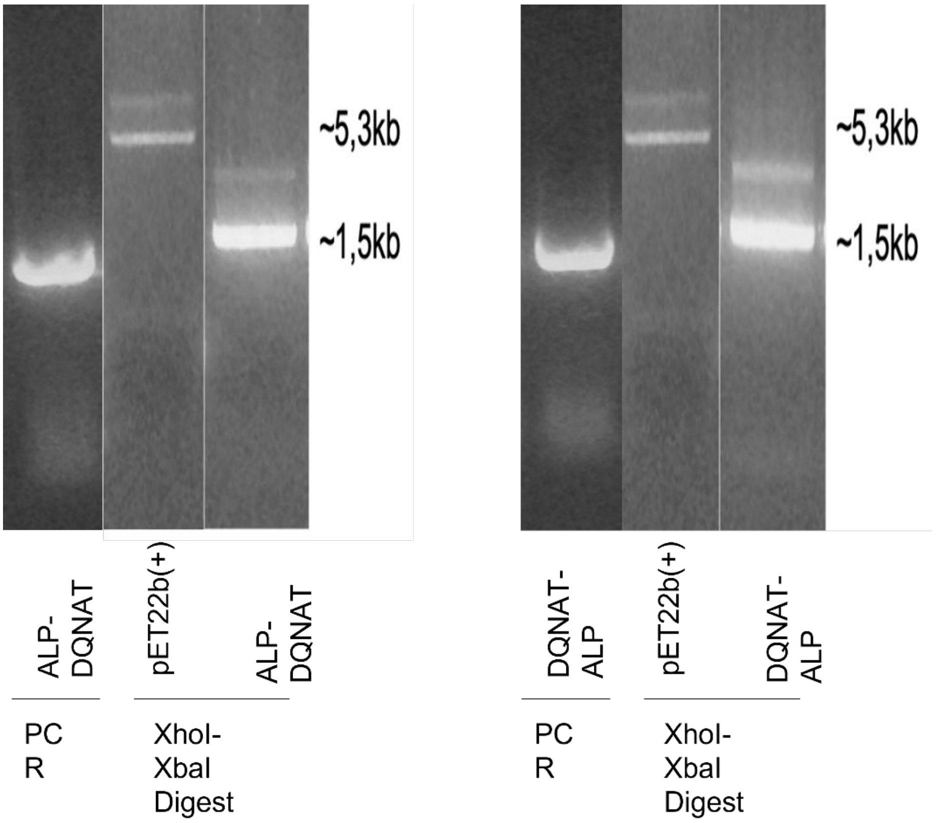

Figure S 2: The cloning steps of constructs for ALP-variants.

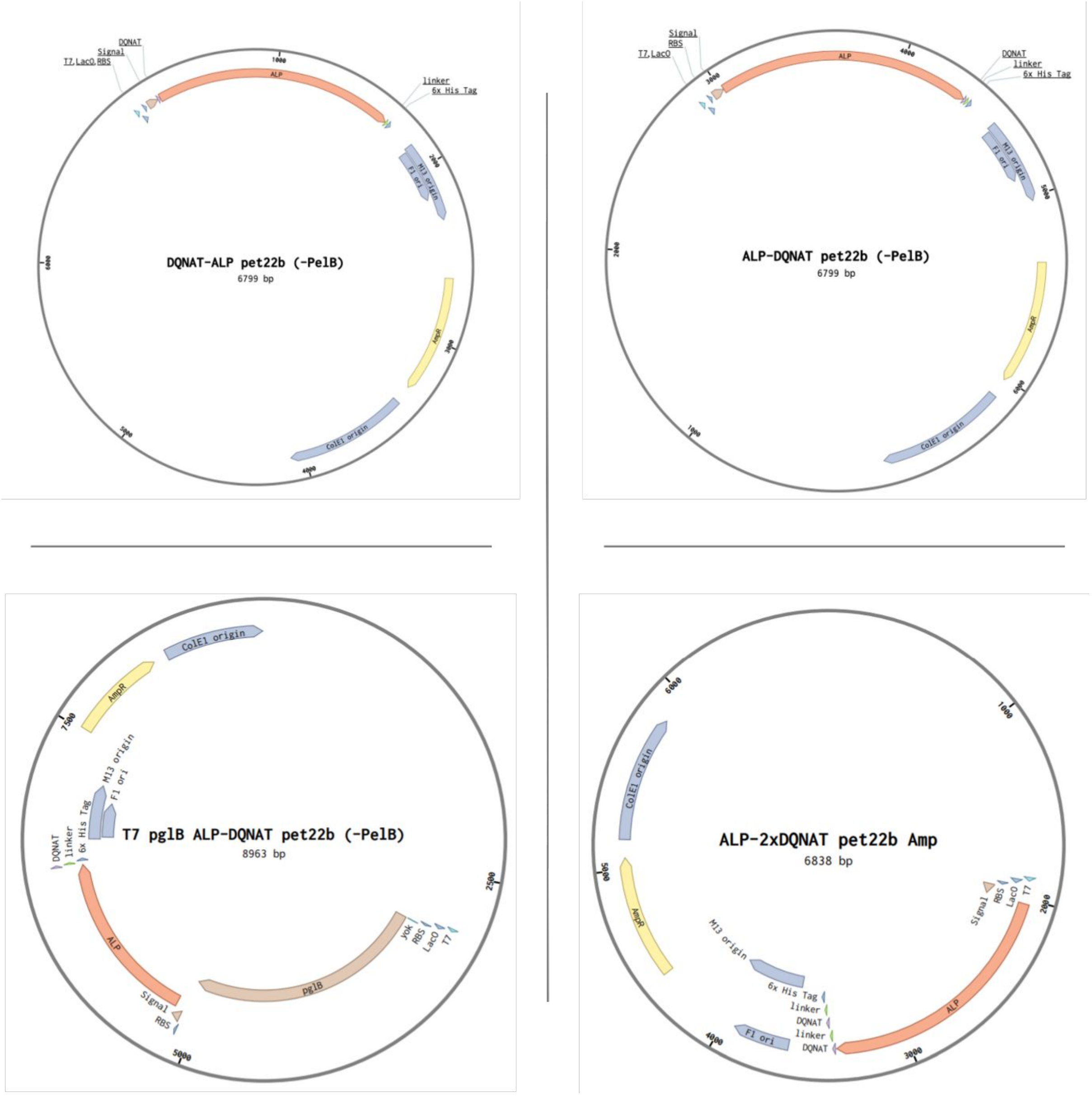

Figure S 3: Plasmids constructed in this study.

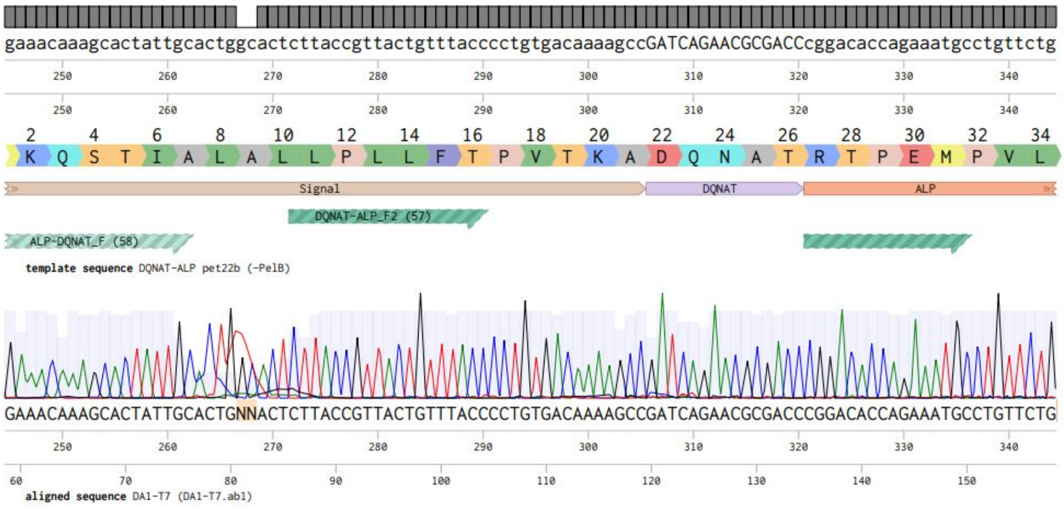

Figure S 4: Sanger sequencing verification results of DQNAT-ALP pET22b

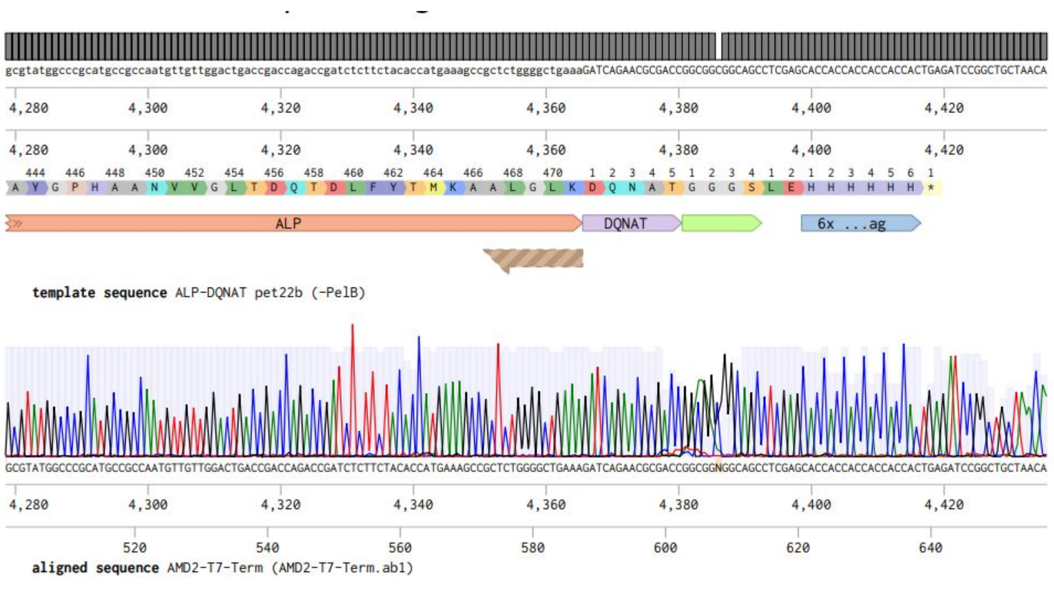

Figure S 5: Sanger sequencing verification result of ALP-DQNAT pET22b

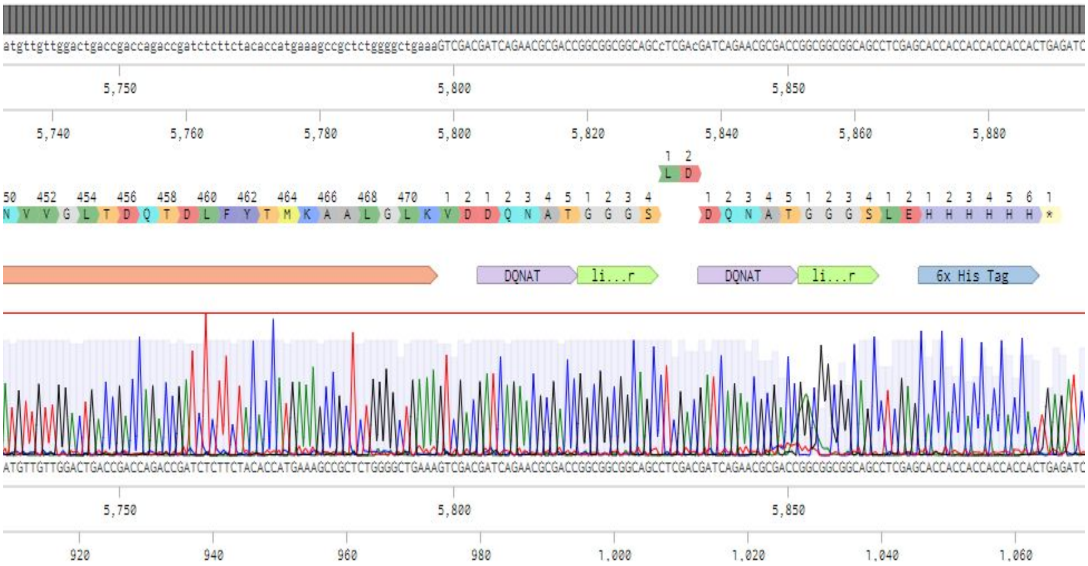

Figure S 6: Sanger sequencing verification result of ALP-DQNAT(x2) pET22b

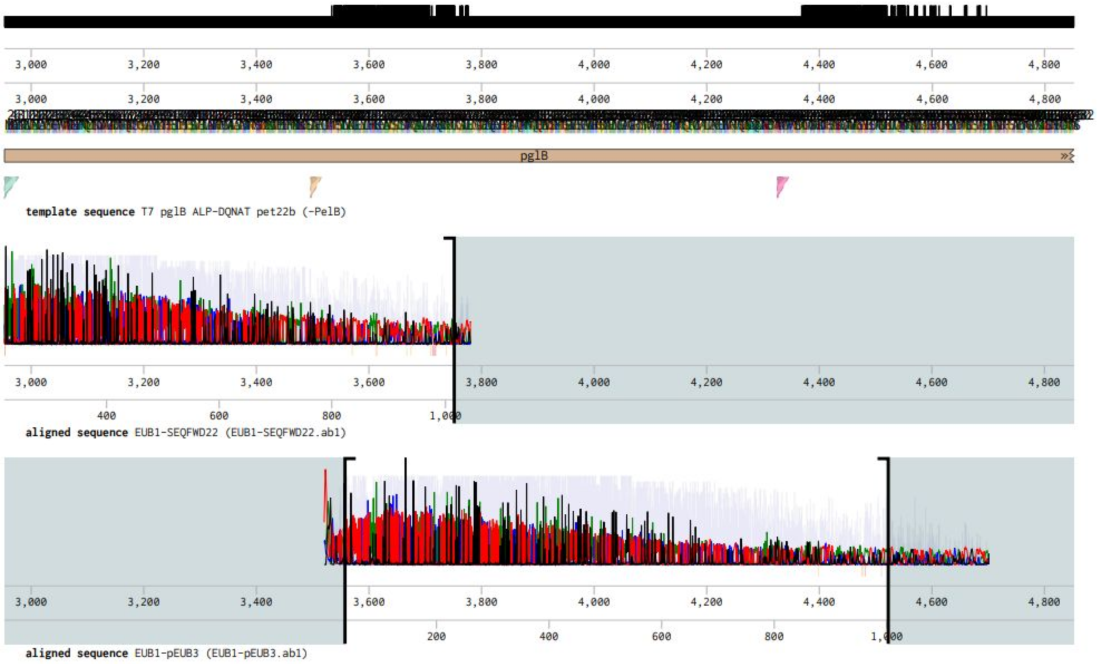

Figure S 7:Sanger sequencing result of pglB insertion into ALP-DQNAT pET22b

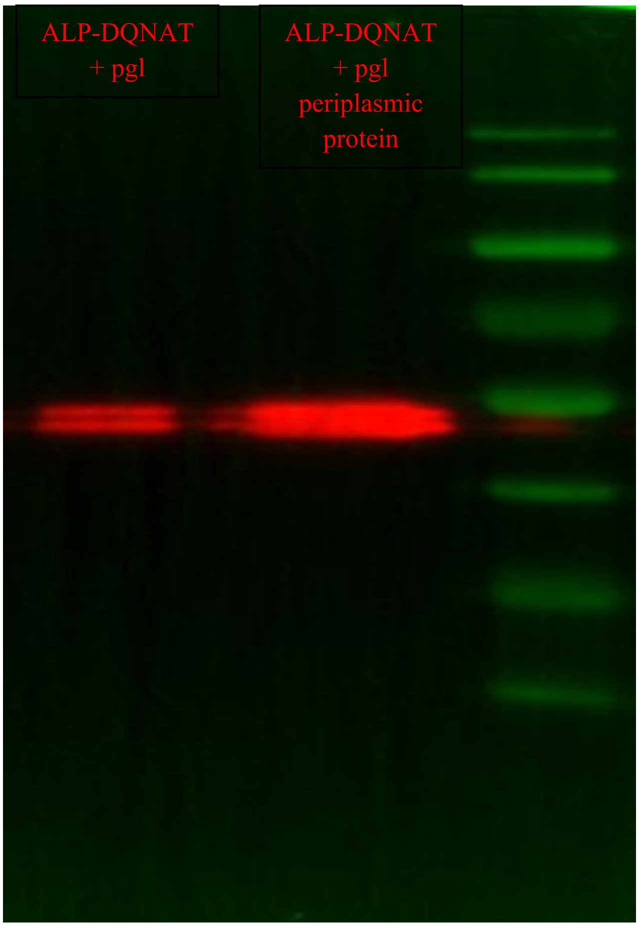

Figure S 8: Western blot result for proteins extracted from periplasmic space.The results showedthat periplasmic protein extraction did not increase theglycosylatedprotein yield and thereforedid not change the glycosylation rate significantly.

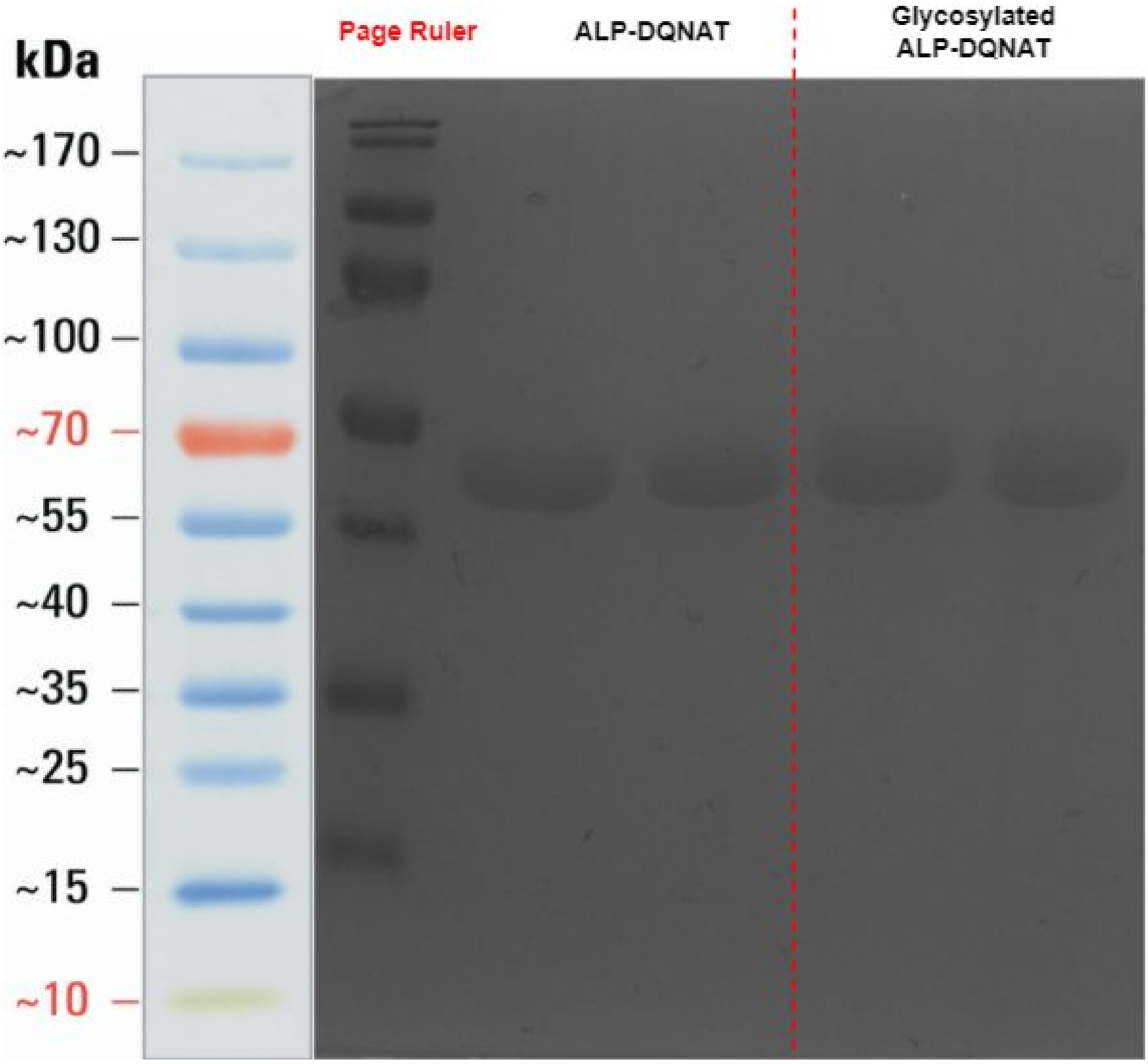

Figure S 9:SDS-PAGE analysis for purified proteins. Results showed that both proteins were purified with high purity.

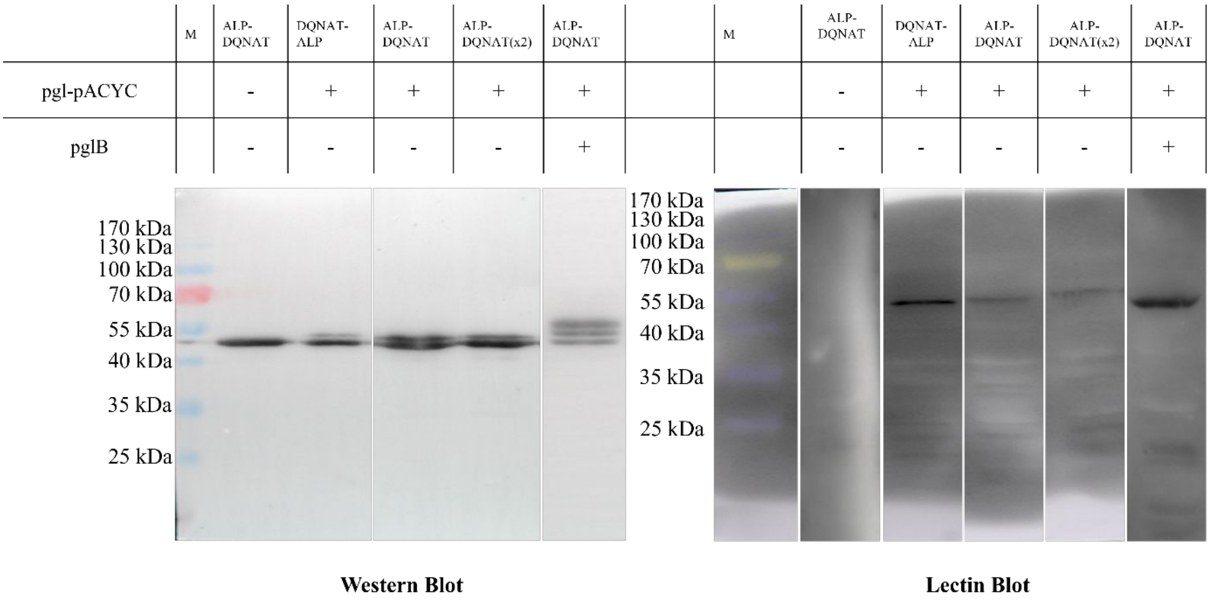

Figure S 10: Full-size images of western blot(Histag Ab) and lectin blot(SBA Antibody) results for ALP-DQNAT variants shown in Figure 1B.

Position	Residue	Score
10	NRA	-0,63298651
45	NII	-0,46195507
64	NYA	-0,41202729
91	NKK	-0,41927758
118	**NGA**	0,20435362
146	**NVS**	0,21998587
182	NAL	-0,39246534
198	NAR	0,012481975
245	**NSV**	0,21858822
251	NQQ	-0,23148619
264	NMP	-0,40149138
279	NII	-0,36998229
290	NPQ	-0,76691008
294	NDS	-0,21727658
314	**NEK**	0,073995766
335	NPC	-0,25937741

362	**NTL**	0,042110277
392	NTK	-0,12752397
405	NSE	-0,27856817
429	NVV	-0,14465687
453	**NAT**	0,37399455

Table S1: Putative N-Linked glycosylation site analysis performed by GlycoPP software. Putative glycosylated aminoacids were underlined and potentially glycosylated sites were written in bold characters. The DQNAT site was shown in blue.

Plasmid Name	Variant	Amino acid Sequence
DQNAT-ALP pET22b	DQNAT-ALP	MKQSTIALALLPLLFTPVTKADQNATRTPEMPVLENRAAQGDITAPGG<br>ARRLTGDQTAALRDSLSDKPAKNIILLIGDGMGDSEITAARNYAEGAG<br>GFFKGIDALPLTGQYTHYALNKKTGKPDYVTDSAASATAWSTGVKTY<br>NGALGVDIHEKDHPTILEMAKAAGLATGNVSTAELQDATPAALVAHV<br>TSRKCYGPSATSEKCPGNALEKGGKGSITEQLLNARADVTLGGGAKTF<br>AETATAGEWQGKTLREQAQARGYQLVSDAASLNSVTEANQQKPLLG<br>LFADGNMPVRWLGPKATYHGNIDKPAVTCTPNPQRNDSVPTLAQMT<br>DKAIELLSKNEKGFFLQVEGASIDKQDHAANPCGQIGETVDLDEAVQR<br>ALEFAKKEGNTLVIVTADHAHASQIVAPDTKAPGLTQALNTKDGAVM<br>VMSYGNSEEDSQEHTGSQLRIAAYGPHAANVVGLTDQTDLFYTMKA<br>ALGLKGGGSLEHHHHHH*

ALP-DQNAT pET22b / T7 pglB ALP DQNAT	ALP-DQNAT	MKQSTIALALLPLLFTPVTKARTPEMPVLENRAAQGDITAPGGARRLTGDQTAALRDSLSDKPAKNIILLIGDGMGDSEITAARNYAEGAGGFFKGIDALPLTGQYTHYALNKKTGKPDYVTDSAASATAWSTGVKTYNGALGVDIHEKDHPTILEMAKAAGLATGNVSTAELQDATPAALVAHVTSRKCYGPSATSEKCPGNALEKGGKGSITEQLLNARADVTLGGGAKTFAETATAGEWQGKTLREQAQARGYQLVSDAASLNSVTEANQQKPLLGLFADGNMPVRWLGPKATYHGNIDKPAVTCTPNPQRNDSVPTLAQMTDKAIELLSKNEKGFFLQVEGASIDKQDHAANPCGQIGETVDLDEAVQRALEFAKKEGNTLVIVTADHAHASQIVAPDTKAPGLTQALNTKDGAVMVMSYGNSEEDSQEHTGSQLRIAAYGPHAANVVGLTDQTDLFYTMKAALGLKDQNATGGGSLEHHHHHH*
ALP-DQNAT(x2) pET22b	ALP-DQNAT(x2)	MKQSTIALALLPLLFTPVTKARTPEMPVLENRAAQGDITAPGGARRLTGDQTAALRDSLSDKPAKNIILLIGDGMGDSEITAARNYAEGAGGFFKGIDALPLTGQYTHYALNKKTGKPDYVTDSAASATAWSTGVKTYNGALGVDIHEKDHPTILEMAKAAGLATGNVSTAELQDATPAALVAHVTSRKCYGPSATSEKCPGNALEKGGKGSITEQLLNARADVTLGGGAKTFAETATAGEWQGKTLREQAQARGYQLVSDAASLNSVTEANQQKPLLGLFADGNMPVRWLGPKATYHGNIDKPAVTCTPNPQRNDSVPTLAQMTDKAIELLSKNEKGFFLQVEGASIDKQDHAANPCGQIGETVDLDEAVQRALEFAKKEGNTLVIVTADHAHASQIVAPDTKAPGLTQALNTKDGAVMVMSYGNSEEDSQEHTGSQLRIAAYGPHAANVVGLTDQTDLFYTMKAALGLKVDDQNATGGGSLDDQNATGGGSLEHHHHHH*

Table S2: Amino acid sequences of ALP variants used in the study.
